# Systematic analysis and expression profiles of *TCP* gene family in Tartary buckwheat (*Fagopyrum tataricum* (L.) Gaertn.) revealed the potential function of *FtTCP15* and *FtTCP18* in response to abiotic stress

**DOI:** 10.1186/s12864-022-08618-1

**Published:** 2022-06-02

**Authors:** Mingfang Yang, Guandi He, Qiandong Hou, Yu Fan, Lili Duan, Kuiyin Li, Xiaoliao Wei, Zhilang Qiu, Erjuan Chen, Tengbing He

**Affiliations:** 1 Agricultural College, Guizhou University, Guiyang, Guizhou, 550025 People’s Republic of China; 2 Big Data Application and Economics College, Guizhou Finance and Economics University, Guiyang, Guizhou, 550025 People’s Republic of China; 3 Key Laboratory of Plant Resources Conservation and Germplasm Innovation in Mountainous Region (Guizhou University), Ministry of Education, Institute of Agro-Bioengineering, College of Life Sciences, Guizhou University, Guiyang, Guizhou, 550025 People’s Republic of China; 4 Agricultural College, Anshun University, AnshunGuihzou, 561000 People’s Republic of China; 5 State Key Laboratory of Functions and Applications of Medicinal Plants, Guizhou Medical University, Guiyang, Guizhou, 550014 People’s Republic of China; 6 Institute of New Rural Development, Guizhou University, Huaxi District, Guiyang City, Guizhou, 550025 People’s Republic of China

**Keywords:** *TCP* transcription factor, Abiotic stress, Tartary buckwheat, *FtTCP15*, *FtTCP18*

## Abstract

**Background:**

As transcription factors, the *TCP* genes are considered to be promising targets for crop enhancement for their responses to abiotic stresses. However, information on the systematic characterization and functional expression profiles under abiotic stress of *TCP*s in Tartary buckwheat (*Fagopyrum tataricum* (L.) Gaertn.) is limited.

**Results:**

In this study, we identified 26 *FtTCP*s and named them according to their position on the chromosomes. Phylogenetic tree, gene structure, duplication events, and cis-acting elements were further studied and syntenic analysis was conducted to explore the bioinformatic traits of the *FtTCP* gene family. Subsequently, 12 *FtTCP* genes were selected for expression analysis under cold, dark, heat, salt, UV, and waterlogging (WL) treatments by qRT-PCR. The spatio-temporal specificity, correlation analysis of gene expression levels and interaction network prediction revealed the potential function of *FtTCP15* and *FtTCP18* in response to abiotic stresses. Moreover, subcellular localization confirmed that *FtTCP15* and *FtTCP18* localized in the nucleus function as transcription factors.

**Conclusions:**

In this research, 26 *TCP* genes were identified in Tartary buckwheat, and their structures and functions have been systematically explored. Our results reveal that the *FtTCP15* and *FtTCP18* have special cis-elements in response to abiotic stress and conserved nature in evolution, indicating they could be promising candidates for further functional verification under multiple abiotic stresses.

**Supplementary Information:**

The online version contains supplementary material available at 10.1186/s12864-022-08618-1.

## Background

Transcription factors usually contain DNA-binding domains, transcription regulation domains, oligomerization sites and nuclear localization signals. Some domains are used to bind DNA, and some are used to activate transcription [1]. A total of 58 transcription factor families have been identified from 156 plant genome sequences [2]. Many studies have shown that identifying key transcription factors provides new insights into how plants respond to abiotic stresses at the cellular and molecular levels [3–8]. Abiotic stress factors or their different combinations can affect the yield and quality of crops on cultivated land worldwide by influencing physiological and biochemical processes within the plant [9–11]. In consideration of global climate change, transcription factors are promising targets for crop enhancement because they are key regulatory switches that control gene expression in various organic and metabolic processes [12].

The transcription factor *TCP* gene family was originally identified and named based on the characteristics of the first three family members of *Zea mays* (L.) Sp. (TB1), *Antirrhinum majus* L. (CYC), and *Oryza sativa* L. (PCF) [13]. The *TCP* gene family contains highly conserved and non-canonical TCP domains with a basic helix-loop-helix structure. These domains play a role in protein-protein dimerization and DNA binding [14, 15]. The *TCP* family is plant-specific and can regulate plant growth, including seed germination and seedling establishment [6], spikelet meristem [16], and branching [17]. Online interaction network prediction shows that the *TCP* gene family also participates in regulating and synthesizing numerous protein complexes involved in various biological processes such as plant hormone pathways, cell life cycles, and environmental stress responses [18]. Many studies have confirmed that the *TCP* family is involved in plant responses to abiotic stresses. When the *OsTCP19* gene is over-expressed in Arabidopsis, it can enhance plant tolerance to drought and heat during seedling and maturity stages [19]. Additionally, down-regulation of *AsTCP14* in the Creeping bentgrass (*Agrostis stolonifera* L.) can improve plant resistance to drought and salt stresses [20]. *TCP* transcription factors inhibit the formation of trichomes on the side of Arabidopsis (*Arabidopsis thaliana* (L.) Heynh.) cotyledon, thus affecting the barrier function of these trichomes against biotic and abiotic stresses on the plant [21].

Buckwheat is a pseudo-cereal crop with a long cultivation history and incomparable nutritional characteristics and is one of the potential research objects to realize the diversification of food resources [22–24]. Buckwheat is cultivated and consumed worldwide and has received widespread attention for its synthesis by many substances with health functions and active medicinal ingredients [24–26]. The buckwheat is usually planted in habitats with low rainfall, low temperatures, and high altitudes [27, 28]. In addition, sufficient light and ultraviolet radiation, for example, high UV-B doses, can affectthe physiological and biochemical processes of plants [29–31], contributing to unusual energy conversion efficiency and plant tissue function [32].To sum up, the growth, yield, and quality of buckwheat are severely threatened by abiotic stress factors or their combinations. To date, many transcription factors in Tartary buckwheat have been well-studied. Some of these transcription factors have been identified to be associated with Al stress [33] and salt stress [34]. However, information on the identification, expression analysis, and potential functions under abiotic stresses of *TCP* transcription factors in Tartary buckwheat is limited.

In this study, the genome-wide identification of 26 *TCPs* in Tartary buckwheat (*Fagopyrum tataricum* (L.) Gaertn.) was carried out. Subsequently, *FtTCP15* and *FtTCP18* were compared with other members of the *FtTCP* gene family in terms of evolutionary relationship, gene structure, conservative motifs, and cis-acting elements. Next, expression profiles of selected 12 *FtTCP* genes under multiple abiotic stresses, including cold, dark, heat, salt, UV, and waterlogging (WL), were analyzed by qRT-PCR, *FtTCP15* and *FtTCP18* were found to be highly expressed under multiple treatments. Correlation coefficient analysis of gene expression levels, tissue-specific expression, and interaction network prediction was conducted. Then, subcellular localization through laboratory methods demonstrated that both *FtTCP15* and *FtTCP18* were localized in the nucleus. Our work provides a comprehensive framework for how *FtTCP*s respond to abiotic stresses and affords valuable clues for further functional verification of *FtTCP15* and *FtTCP18* as transcription factors in Tartary buckwheat to improve the adaptability under multiple abiotic stresses.

## Results

### The 26 *FtTCP* genes identified in the Tartary buckwheat genome are unevenly distributed on 8 chromosomes

**Fig. 1:**
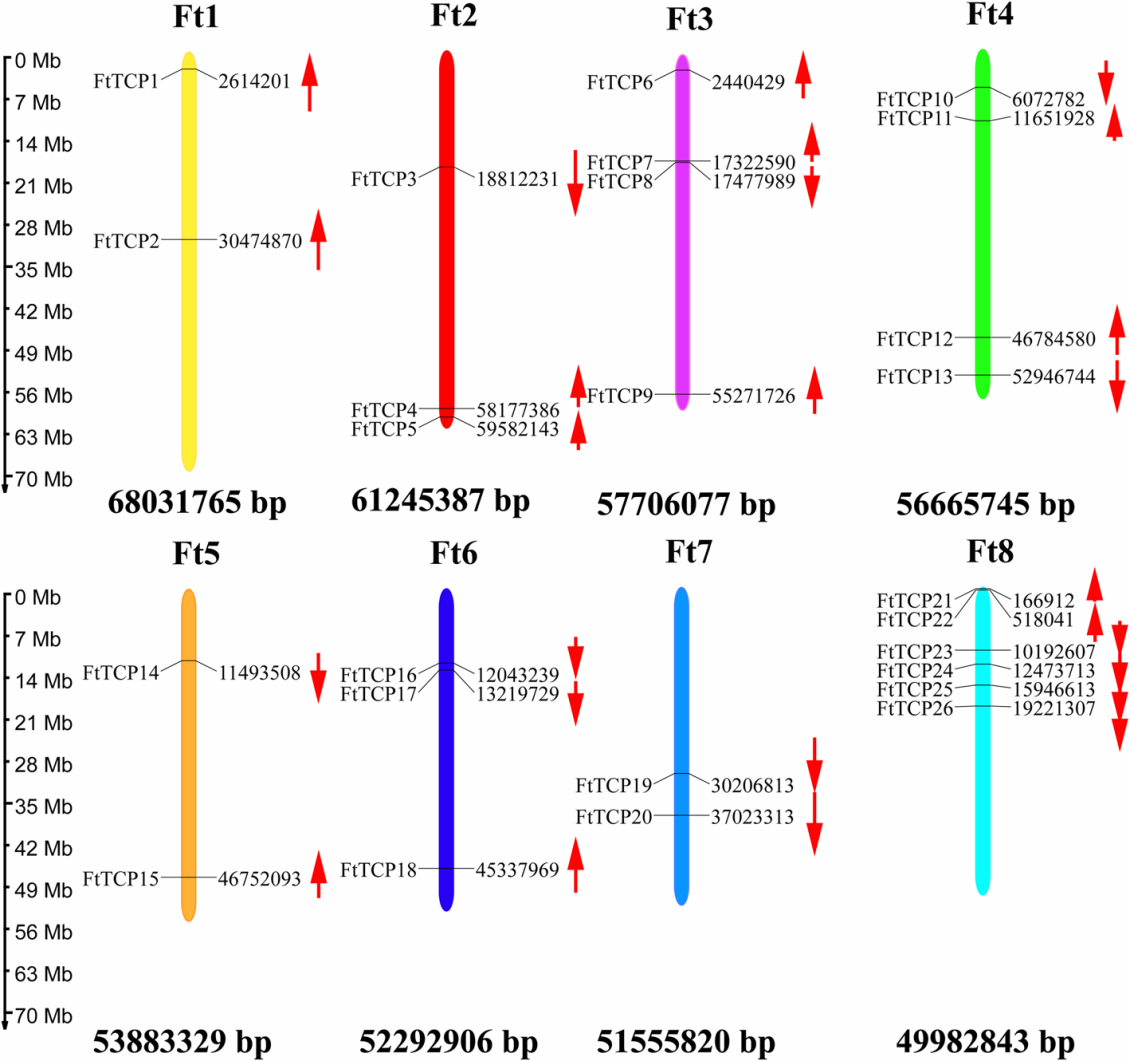
Schematic representations of the chromosomal distribution of the 26 *TCP*s in Tartary buckwheat. The position of the gene on the chromosome and the corresponding base pair position are marked on the left and right sides. The chromosome number and chromosome full-length base pair are labeled above and below each chromosome. The upward red arrow indicates that the gene has a reverse chain on the chromosome, and the downward red arrow indicates that the gene has a forward chain on the chromosome

In this paper, 26 *FtTCP*s from the Tartary buckwheat genome were identified and named from *FtTCP1* to *FtTCP26* according to their position on the chromosome (Fig. 1). The distribution and arrangement information of genes on chromosomes in the latest Tartary buckwheat gene bank were used for chromosome distribution maps. Figure 1 shows that the 26 *FtTCP* genes of Tartary buckwheat are unevenly distributed on 8 chromosomes, with 2 to 6 genes on each chromosome. The Ft8 has the largest number of *FtTCP* genes (6 genes), while Ft1, Ft5, and Ft7 have the least number of *FtTCP* genes (2 genes). There are 3 genes on Ft2 and Ft6, and 4 genes on chromosomes Ft3 and Ft4. Additionally, the two genes on Ft1 are on the reverse chain, and both the two genes on Ft7 are on the forward chain. The genes on the remaining chromosomes are also in forward or reverse chains.

Table S1 shows detailed information about these *FtTCP*s, including the gene name, sequence ID, group, length of protein (PL), molecular weight (MW), the isoelectric point (pI), genome location, and predicted subcellular location (W). The length of the identified *FtTCP* gene-encoded proteins ranges from 176 aa (*FtTCP2*) to 475 aa (*FtTCP3*), with an average of 285.8 aa. The range of MW is from 19.17 kDa (*FtTCP2*) to 51.04 kDa (*FtTCP3*), with an average of 31.09 kDa. The theoretical pI also differs greatly from 4.93 (*FtTCP20*) to 9.71 (*FtTCP19*) with an average of 7.05. According to the subcellular localization prediction, 21 *FtTCP* genes have the highest probability of being in the nucleus, and 5 *FtTCP* members are only located in the nucleus. Additionally, the group and chromosome location of the identified *FtTCP* genes were provided.

### The phylogenic relationship and conserved features predict the potential functions of *FtTCP15* and *FtTCP18*

**Fig. 2:**
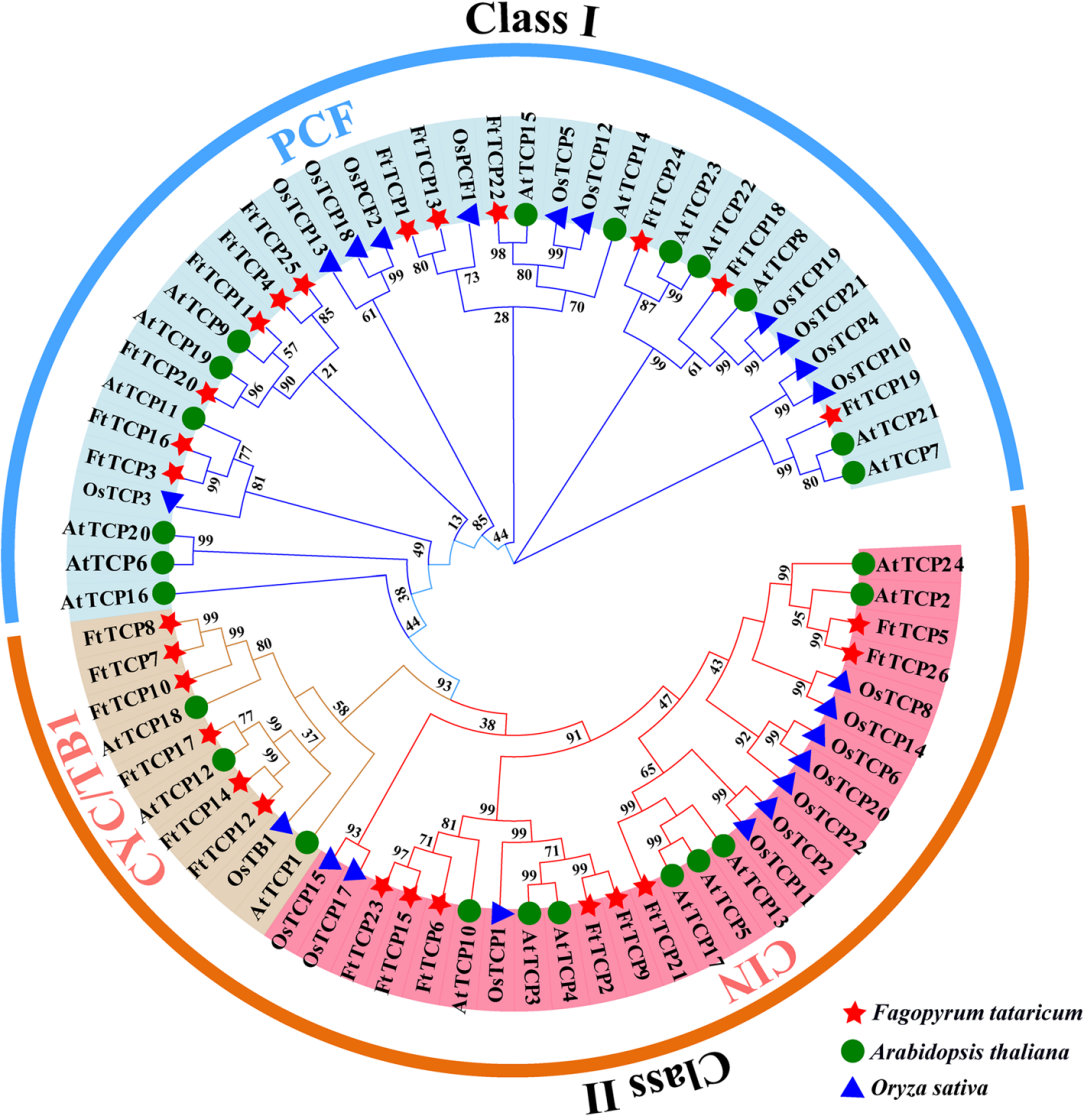
Phylogenetic analysis of TCP proteins among Arabidopsis thaliana, rice, and Tartary buckwheat. The phylogenetic tree was constructed with the MEGA 7.0 Software, using parameters as bootstrap values (1000 replicates) and the Poisson model. The numbers on each branch line represent bootstrap values. All the *TCP*s are divided into three groups: PCF (belonging to Class I), CIN and CYC/TB1 (belonging to Class II). Tartary buckwheat is shown with red five-pointed star icons, Arabidopsis thaliana is shown with green circle icons, and rice is shown with blue triangle icons

In order to understand the phylogenetic relationship among *TCP* genes of Tartary buckwheat, Arabidopsis, and rice, the full-length sequence of *TCP* proteins with known functions was selected to draw a rootless phylogenetic tree (Fig. 2). The numbers of proteins from Tartary buckwheat (FtTCP), Arabidopsis (*Arabidopsis thaliana* (L.) Heynh.) (AtTCP), and rice (*Oryza sativa* L.) (OsTCP) were 26, 24, and 22, respectively. The names used for the *AtTCP* in Arabidopsis and *OsTCP* in rice were based on the previous study [35]. The result shows that all 72 *TCP* members are classified into Class I and Class II subfamilies. Class I contains the PCF group, and Class II contains two subclades (CYC/TB1 and CIN). The phylogenetic tree shows that 36 *TCP*s belong to the PCF clade, 26 to CIN, and 10 to CYC/TB1. The results show that *AtTCP*s and *OsTCP*s in the phylogenetic tree fall into the same Class or subclade consistent with the finding in the previous study [35]. All the 26 *FtTCPs* are classified into three clades, with 12, 6, and 8 in PCF, CYC/TB1 and CIN, respectively. The distribution of the *TCP* genes in each subfamily is random and uniform. For example, there is only 1 from rice, 6 from Tartary buckwheat, and 3 from Arabidopsis among the 10 CYC/TB1 *TCP* genes.

As shown in Fig. 2, most Arabidopsis class II *TCP* genes (in the CYC/TB1 group) have one or more counterparts in Tartary buckwheat and rice. *OsTCP1, FtTCP12*, and *FtTCP14* correspond to *AtTCP1*. *FtTCP17* correspond to *AtTCP12*. *FtTCP10*, *FtTCP7*, and *FtTCP8* correspond to *AtTCP18*. *FtTCP6*, *FtTCP15*, and *FtTCP23* correspond to *AtTCP10*. However, *AtTCP6*, *AtTCP16*, and *AtTCP20* have a separation position with no close homology in Tartary buckwheat, implying a lineage-specific gene loss in Tartary buckwheat. A similar result of *AtTCP16* was also observed in petunia [36] and tomatoes [37]. In particular, *FtTCP15* and *FtTCP23* are isolated from each other, and their evolutionary relationships are closely related to *FtTCP6* and *AtTCP10*. In contrast, *FtTCP18* is closely related to *AtTCP8*, *OsTCP19*, and *OsTCP21*, indicating that these genes in the same cluster may have similar functions in response to abiotic stresses. Therefore, *FtTCP15* and *FtTCP18* may have the same function as *AtTCP10* and *OsTCP19*.

**Fig. 3:**
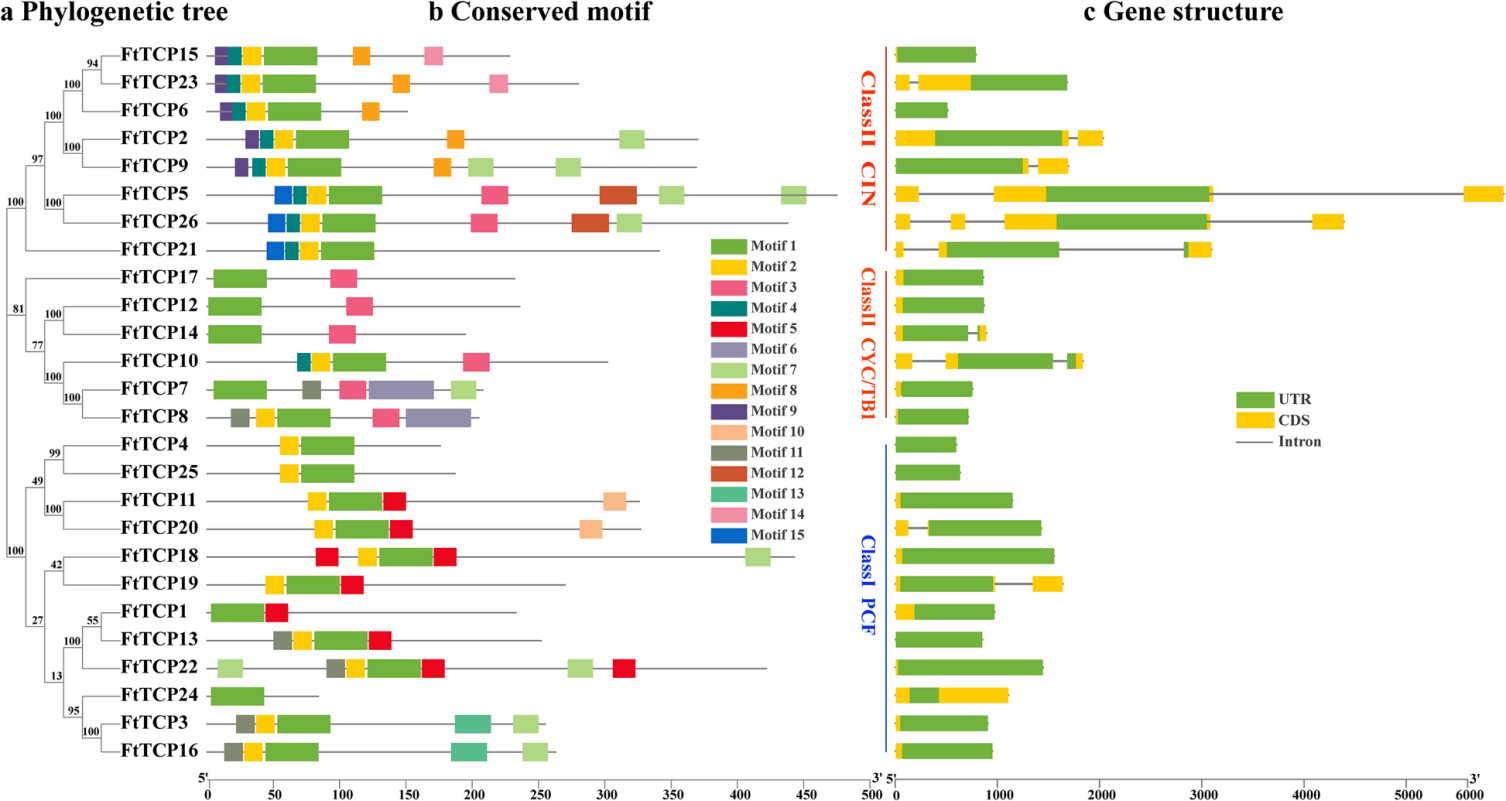
Phylogenetic relationship, gene structure, and architecture of conserved protein motifs in *TCP* genes from Tartary buckwheat. **a** The phylogenetic tree is constructed based on the full-length sequences of Tartary buckwheat TCP proteins using Geneious R11 software. **b** Amino acid motifs in the Tartary buckwheat are represented by 15 colored boxes, and the relative protein lengths are indicated by black lines. **c** The exon–intron structure of Tartary buckwheat *TCP* genes. Exons, introns, and untranslated regions (UTRs) are indicated by blue rectangles, black lines, and green rectangles, respectively. The unit of the bottom ruler is bp

As shown in Fig. 3, the structural features and conserved motifs have been performed to further study *TCP* genes. The analysis provides a reference to analyze the evolutionary relationship and the structural diversity of members in each subfamily and to fully understand conserved motifs and gene structure of the *TCP* family. As shown in Fig. 3b, there are 15 conserved motifs (motif 1-motif 15) of the FtTCP protein predicted by the online MEME analysis tool. The different colored boxes in the legend correspond to distinct motifs. The logo and name of the query motifs are displayed in Table S2. Motif 1 and Motif 2 are present in nearly all FtTCP proteins and Motif 1 is included in every protein. However, other motifs exist in less than half of the proteins. Many motifs, such as motifs 6, 9, 14, and 15, shared by the class II (CYC/TB1 and CIN) TCP family, are restricted to specific groups. The CIN group genes only share the last three motifs. Additionally, Motif 13 only exist in *FtTCP3* and *FtTCP16* (belonging to PCF Group), and Motif 6 is only found in *FtTCP7* and *FtTCP8* (belong to CYC/TB1). *FtTCP24* only has Motif 1.

The structural features of the *FtTCP* gene family, including the arrangement and quantity characteristics of exons and introns, are shown in Fig. 3c. Gene family members belonging to the same clade have similar numbers and organization of exons/introns. The gene structure of the *FtTCP* family is characterized by a small number of introns. There are 16 *TCP* gene CDS with no introns (account for 62%). Most of them are from Class I (PCF), and 6 members (*FtTCP6*, *FtTCP7*, *FtTCP8*, *FtTCP12*, *FtTCP15*, and *FtTCP17*) are from Class II. Among the remaining 10 genes with introns, 8 belong to Class II (6 belong to CIN Group, 2 belong to CYC/TB1 Group). The CIN group contains the most members of introns compared with the other two groups. The *FtTCP5* belonging to CIN has the longest exon. Moreover, the number of introns contained in the remaining 10 genes ranges from 1 to 3, indicating little difference among these members. A total of 22 *FtTCP* members contain exons, accounting for 84%. The genes with exons in the Arabidopsis *TCP* family account for about 82% [35]. The results indicate that buckwheat and Arabidopsis have similar gene conservatism. Both *FtTCP15* and *FtTCP18* have no introns, indicating that they are highly conservative in response to abiotic stress. *FtTCP15* contains Motifs 1, 2, 8, 9, 14, 15; *FtTCP15* contains 2 Motif 5, and Motifs 1, 2, 7. Their specific conserved motifs can be used as the targets for further studies on the molecular mechanisms related to the stress response.

### Segmental duplications are the main force in the molecular evolution of *FtTCP* gene amplification

**Fig. 4:**
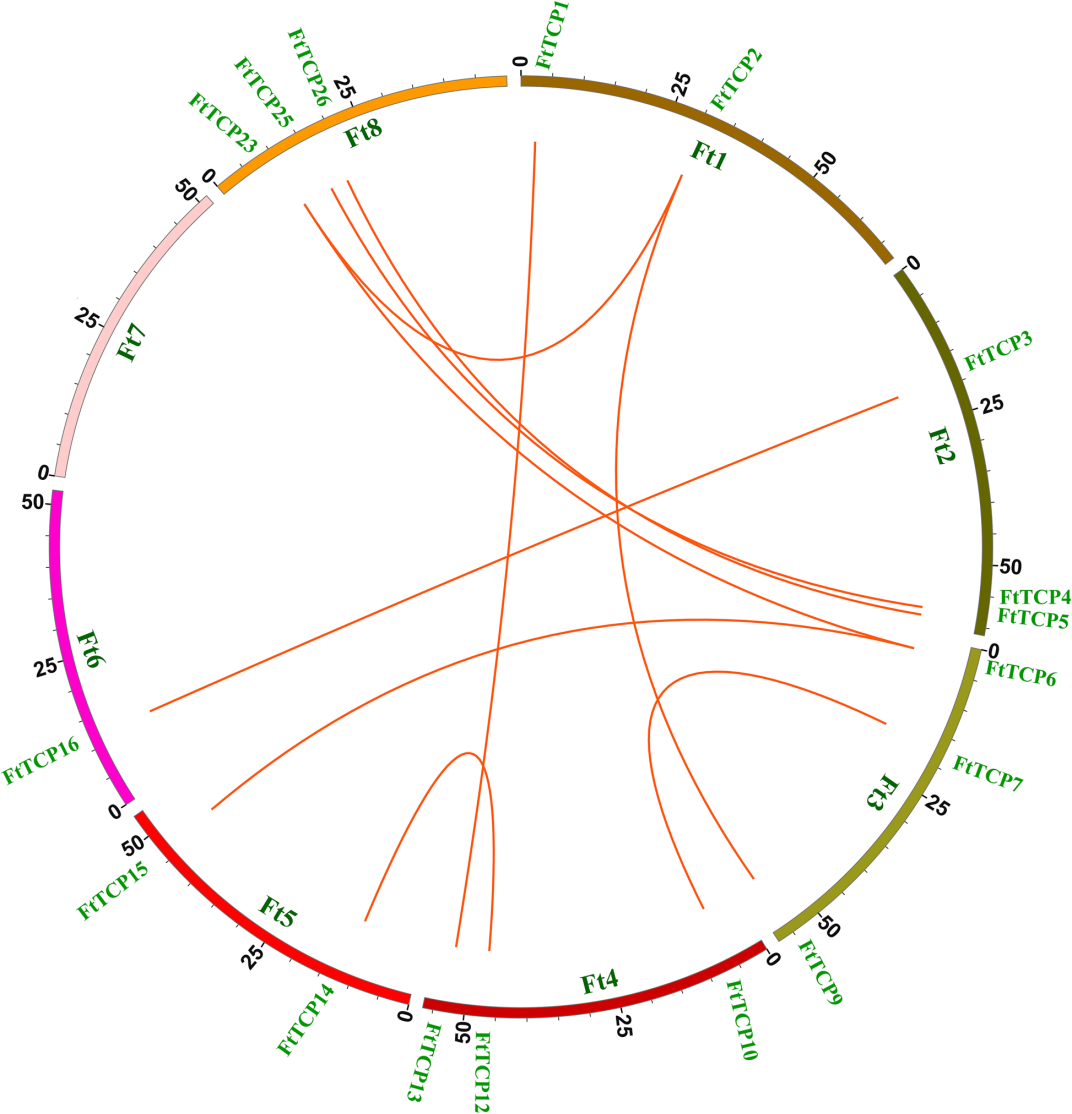
Schematic representations of the chromosomal distribution and intrachromosomal relationships of *TCP* genes in Tartary buckwheat. Colored lines indicate duplicated *TCP* gene pairs in the Tartary buckwheat genome. The chromosome number is indicated along each chromosome

Gene duplication is the main force that promotes the amplification of gene families and generates new functions of genes. The analysis of replication events (Fig. 4) shows that only segmental duplication events happen in the *FtTCP* gene family of the Tartary buckwheat genome, and there are no tandem duplication events. As shown in Fig. 4, there are 10 pairs of segment duplication events in the *FtTCP* gene family. Ft8 and Ft3 have 4 segmental duplication gene pairs. There are 3 segmental duplication gene pairs that occur on Ft1, Ft2, and Ft4, simultaneously. The remaining Ft5 and Ft6 have 2 and 1 segmental duplication gene pairs, respectively. However, no segmental duplication gene pairs occur on the Ft7. As for the subfamily, there are 6 repeated events in Class I (PCF) subfamily. A total of 10 segmental duplication events occurred in CIN subfamily with the highest number, and 4 segmental duplication events occurred in the CYC/TB1 subfamily. There are 14 segmental duplications events in Class II (CIN and CYC/TB1) in all. Segment duplication events can promote the evolution of the *FtTCP* gene family. Intraspecific collinearity was found between *FtTCP15* and *FtTCP6*, but no intraspecific collinearity was found for *FtTCP18*.

**Fig. 5:**
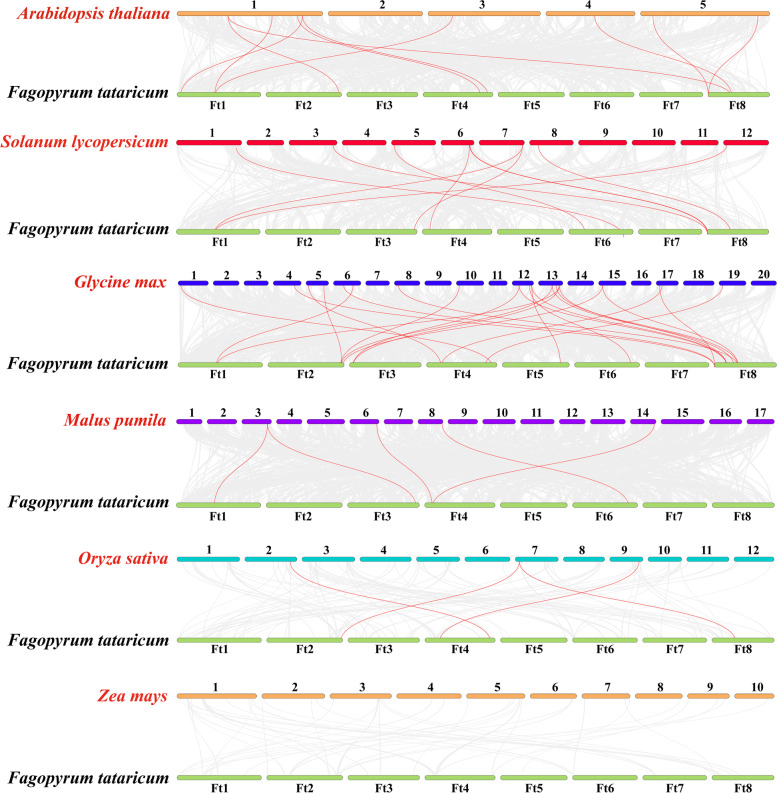
Syntenic analysis of the *TCP* genes between Tartary buckwheat and six representative plants. Gray lines in the background indicate col-linear blocks within Tartary buckwheat and other plant genomes, while red lines highlight synthetic *TCP* gene pairs. The chromosome number is indicated above or below each chromosome

In order to further explore the interspecific collinearity of the *TCP* gene family between Tartary buckwheat and other species, a comparison system diagram between Tartary buckwheat and other six plants was constructed (Fig. 5). The six plants are Arabidopsis (*Arabidopsis thaliana* (L.) Heynh.), apple (*Malus pumila* L.), soybean (*Glycine max* (L.) Merr.), rice (*Oryza sativa* L.), maize (*Zea mays* (L.) Sp.), and tomato (*Solanum lycopersicum* (L.) Mill.). The collinearity relationship among *TCP* genes in different plants is shown in Fig. 5. The *FtTCP* genes have different degrees of collinear relationship with other plants. Soybean has the most homologous pairs, with 24 pairs, followed by Arabidopsis, tomato, apple, and rice, with 10, 9, 5, and 4 homologous pairs, respectively. There is no syntenic relationship between the Tartary buckwheat and *Zea mays* (L.) Sp.. There are 2 orthologous gene pairs between soybean and Tartary buckwheat distributed on Ft1 (*FtTCP2*), 4 on Ft2 (3 pairs on *FtTCP4*; 1 pair on *FtTCP5*), 3 on Ft3, 4 on Ft4, 1 on Ft5 and Ft6, and 10 on Ft8. The Ft4 chromosome of selected plants, except the *Zea mays* (L.) Sp., contains one or more gene pairs.

Furthermore, some genes have more than one pair of collinearity, indicating their important role in evolution. For example, *FtTCP2* (classified into the CIN group) in Tartary buckwheat has two homologous gene pairs with Arabidopsis, tomato, and soybean. The synteny analysis between soybean and Tartary buckwheat reveals that 16 out of 24 orthologous gene pairs are classified into the CIN group, 7 are classified into the PCF group, and only 1 belongs to CYC/TB1. Additionally, the *FtTCP15* located on Ft5 has only one interspecific collinearity gene pair with soybean, and *FtTCP18* located on Ft6 has one pair with tomato, soybean, and apple, respectively, suggesting that they are relatively conservative during evolution.

### Analysis of cis-acting elements of *FtTCP*s reflects their potential function in stress response

**Fig. 6:**
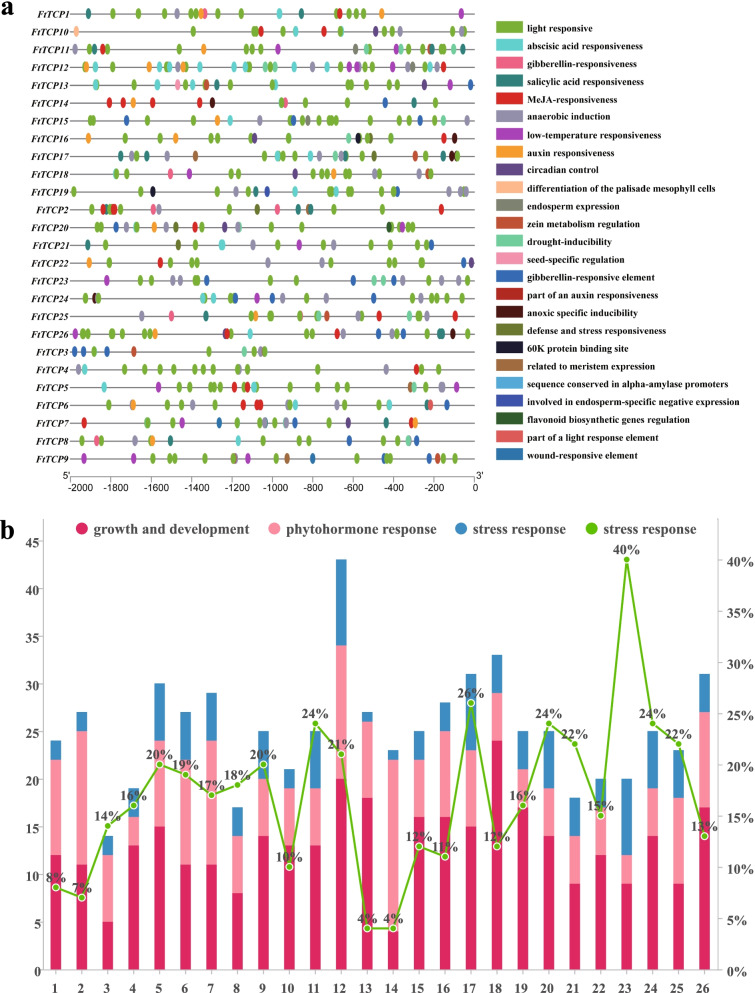
Predicted cis-elements in the promoter regions of the Tartary buckwheat. **a** The sequences within 2000 bp upstream of the *TCP* genes are analyzed. The scale bar at the bottom indicates sequence length. Different cis-elements are labeled by rectangles with different colors based on the legend at the bottom. **b** Promoters are divided into three categories: growth and development, phytohormone response, and stress response. The bar graphs represent the number of the three types in each gene. Red represents growth and development, pink represents phytohormone response, and blue represents stress response. The green broken line graph represents the percentage of stress response in each gene

Cis-acting elements refer to specific DNA sequences in series with structural genes and bind sites for transcription factors. Cis-acting elements regulate the precise initiation and transcription efficiency of gene transcription by binding to transcription factors. In this work, an upstream 2000 bp region was selected to analyze the cis-elements of the *FtTCP* genome sequence (Fig. 6; Table S3; Table S4).

The results show that the cis-acting elements concerning growth and development are the most (328, about 51%). The light-responsive elements (G-box: TACGTG; GT1-motif: GGTTAA; ATC-motif: AGC-TATCCA; AE-box: AGAAACAA; Box 4: ATTAAT; et.al.) are the most in each gene. There are 296 light-responsive elements, accounting for 46%. There are many kinds of elements related to phytohormone response, such as ABRE (ACGTG; CGTACGTGCA), abscisic acid (ABA) (65 in total), Me-JA (CGTCA-motif; TGACG-motif) (60 in total), gibberellin (TATC-box: TATCCCA) (35 in total), and auxin (Aux RR-core: GGTCCAT) (18 in total), which respond to the stress after being induced by adversity factors [38, 39]. At last, multiple abiotic stress-related promoters were explored, such as those with anaerobic induction (ARE: AAACCA) (61 in all genes), anoxic specific inducibility (GC-motif: CCCCCG) (6 in all genes), low-temperature responsiveness (LTR: CCGAAA) (19 in all genes), drought-inducibility (MBS: CAACTG) (17 in total), stress responsiveness (TC-rich repeats: ATTCTCTAAC) (6 in total), and wound-responsiveness (WUN-motif: AAATTTCCT) (2 in total). The sum of stress-related promoters is 105 (about 17%) (Table S4). It is assumed that these two factors (anaerobic induction and anoxic specific inducibility) are important for the response to abiotic stress. All *FtTCP*s are found to have light-responsive elements, and *FtTCP15* and *FtTCP18* have 15 and 11 light-responsive cis-elements, respectively (Table S4). Most cis-elements of *FtTCP15* and *FtTCP18* are related to growth and development.

### The response of the *FtTCP* family to abiotic stress is spatio-temporal, and the interaction network is predicted

**Fig. 7:**
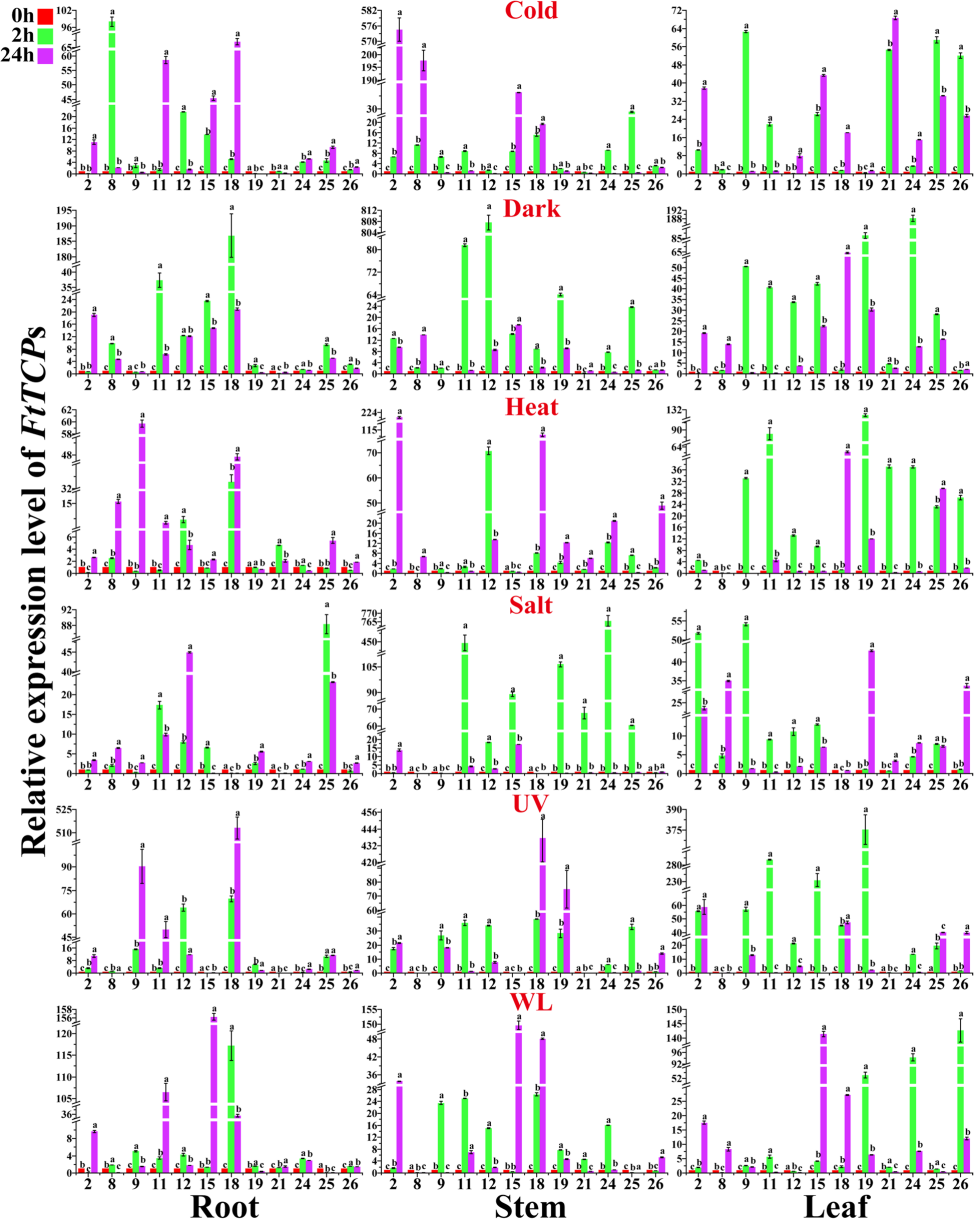
Gene expression profiles of the TCP gene family in Tartary buckwheat under different treatments. The abiotic stress including Cold, Dark, Heat, Salt, UV, and Waterlogging (WL) stress. The plant samples were collected in the six-week-old Tartary buckwheat seedling stage, after being treated for 2 and 24 h under every treatment. Data represent means (± SE) of three biological replicates. Vertical bars indicate standard deviations. The legend in the upper left corner of the image shows the processing time in different colors (0 h, 2 h, 24 h). Different letters (a, b, c) indicate significant differences in terms of different times using Duncan's multiple range test (*P* < 0.05; *n* = 3) by SPSS. The number on the horizontal axis represents the corresponding gene name, for example, 2 represents *FtTCP2*. The chart is constructed by the Origin 8.0 software

A total of 12 *FtTCP*s were selected randomly for expression analysis under multiple abiotic stresses, including cold, dark, heat, salt, UV, and WL. A histogram of the expression of these genes in different tissues after stress at different time points was drawn, and correlation analysis was performed. As shown in Fig. 7 and Figure S1, the expression patterns of *FtTCP*s under different abiotic stress conditions have time and space specificity, and many genes have obvious up-regulation or down-regulation trends.

*FtTCP11* (24 h, root), *FtTCP15* (24 h, root, stem, leaf), *FtTCP18* (2 h, root, stem), *FtTCP24* (2 h, leaf), and *FtTCP26* (2 h, leaf) were significantly up-regulated in WL treatment. Expression levels of *FtTCP18* (24 h, root, stem), *FtTCP11* (2 h, leaf), *FtTCP15* (2 h, leaf), and *FtTCP19* (2 h, leaf) were increased significantly in the UV environment. Expressions of *FtTCP11* (2 h, stem), *FtTCP24* (2 h, stem) and *FtTCP25* (2 h, root) were significantly up-regulated in the salt environment. Additionally, *FtTCP2* (24 h, stem) was up-regulated under cold and heat stress treatments. *FtTCP8* (24 h, stem) was also up-regulated in cold treatment. In summary, 4 *FtTCP*s (*FtTCP11*, *FtTCP15*, *FtTCP18*, and *FtTCP26*), 4 *FtTCP*s (*FtTCP11*, *FtTCP15*, *FtTCP18*, and *FtTCP19*), 3 *FtTCP*s (*FtTCP11*, *FtTCP24*, and *FtTCP25*), 2 *FtTCP*s (*FtTCP12* and *FtTCP18*), 2 *FtTCP*s (*FtTCP2* and *FtTCP8*), and 3 *FtTCP*s (*FtTCP2*, *FtTCP18*, and *FtTCP19*) were restricted by WL, UV, salt, dark, cold, and heat stresses, respectively. Among them, 5 *FtTCP*s (*FtTCP8*, *FtTCP12*, *FtTCP24*, *FtTCP25*, and *FtTCP26*) were activated by the specific stress. Several genes were significantly up-regulated in different stress environments at two time points of stress and in three different tissues. The expression profiles of the selected genes under abiotic stress revealed that *FtTCP18* was generally responsive to different abiotic stresses, and *FtTCP15* was responsive to WL, UV, and salt stress. We are interested in exploring the molecular mechanisms of *FtTCP15* and *FtTCP18* in response to abiotic stresses. Therefore, we conducted a comprehensive study on the evolution of the *FtTCP* gene family to explore the particularity of *FtTCP15* and *FtTCP18*.

**Fig. 8:**
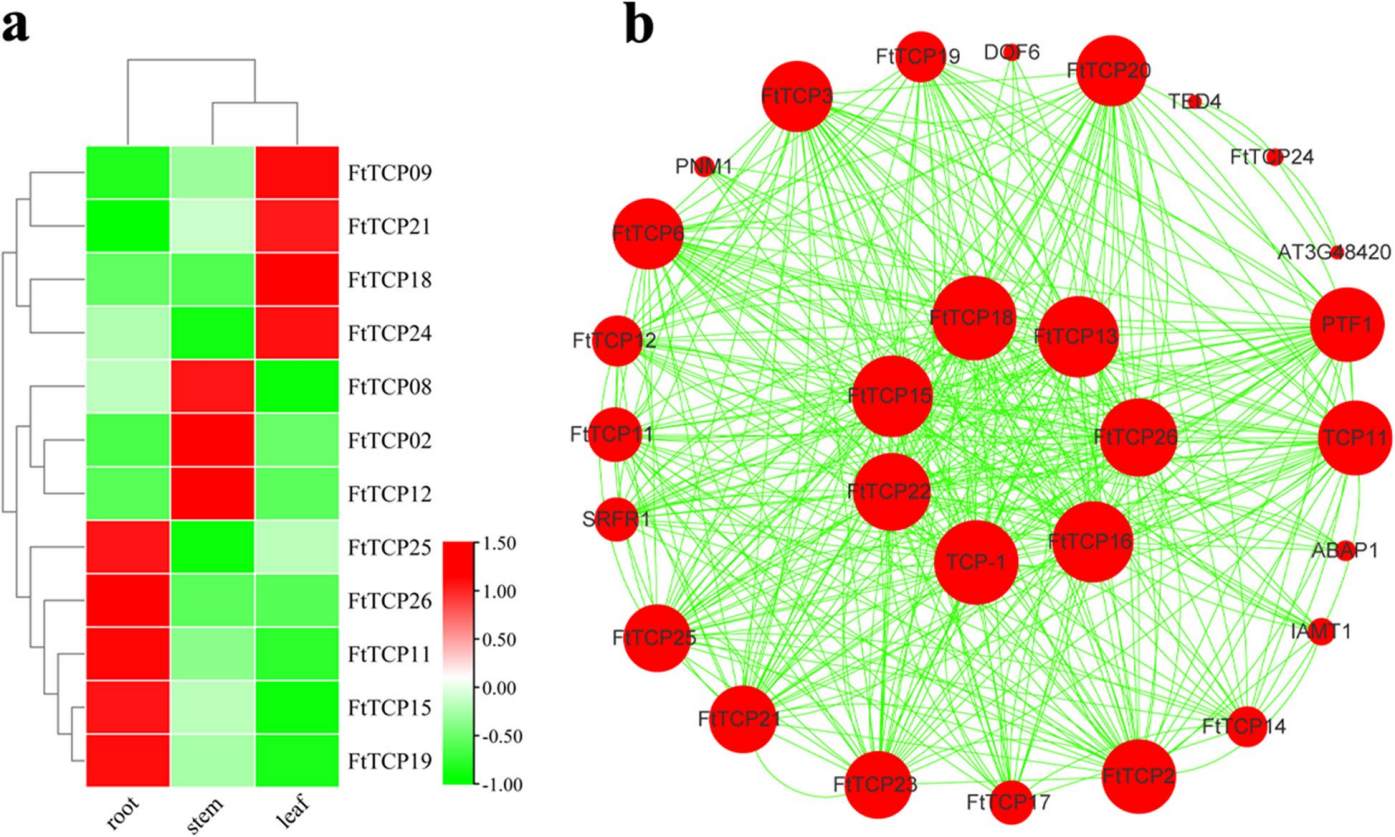
Tissue-specific expression patterns and prediction of interaction network of *FtTCP*s. **a** Tissue-specific expression pattern of 12 selected *FtTCP*s in Tartary buckwheat at the six-week-old seedling stage under natural conditions. The tissues include the root, stem, and leaf. One expression value is shown for each gene in different tissues under natural conditions. The values represent the average expression value of three replicates. **b** Prediction of interaction network among TCP genes. The green line indicates the interaction relationship between genes. The red circle represents the gene that interacts with other TCPs. The size of the circle represents the number of interaction relationships. Greater circles size, mean more interaction times

The correlation analysis of gene expression under different abiotic stress conditions (Figure S1) demonstrates that the maximum correlation coefficient is 0.81 (between *FtTCP2* and *FtTCP8*), and the minimum is -0.09 (between *FtTCP8* and *FtTCP9*). These correlation coefficients prove that these genes have a certain connection when performing their functions. *FtTCP15* and *FtTCP18* are representatives in expression profile analysis under several stresses. A basic overview of multiple potential regulatory mechanisms of *FtTCP*s can be obtained through various expression patterns in different organizations. In addition, tissue-specific expression analysis of the selected genes in Tartary buckwheat was conducted in the seedling stage (six-week-old) under natural conditions. As shown in Fig. 8a, *TCP* genes of Class I are highly expressed in the leaf and root. For example, *FtTCP18* and *FtTCP24* are highly expressed in the leaf, while *FtTCP11* and *FtTCP19* are highly expressed in the root. The genes from Class II are expressed in all tissues. A particular concern is that *FtTCP15* is highly expressed in the root, while the *FtTCP18* is highly expressed in the leaf, indicating their different functions in responding to stresses in different tissues.

Interaction network prediction is significant for studying the function of genes. Interaction prediction data were obtained from STRING (https://string-db.org/) and visualized using Cytoscape software (Table S5; Table S6). As shown in Fig. 8b, the size of the red circle can intuitively represent interaction times between proteins. The FtTCP24 alone interacts with the TBD4 and AT3G48420, indicating that FtTCP24 may have a different function. FtTCP15 and FtTCP18 interact with multiple proteins in the interaction network, and the number of FtTCP15 and FtTCP18 interacting with other proteins is 41 and 44, respectively (Table S6).

### Subcellular localization showed that *FtTCP15* and *FtTCP18* localized in the nucleus

**Fig. 9:**
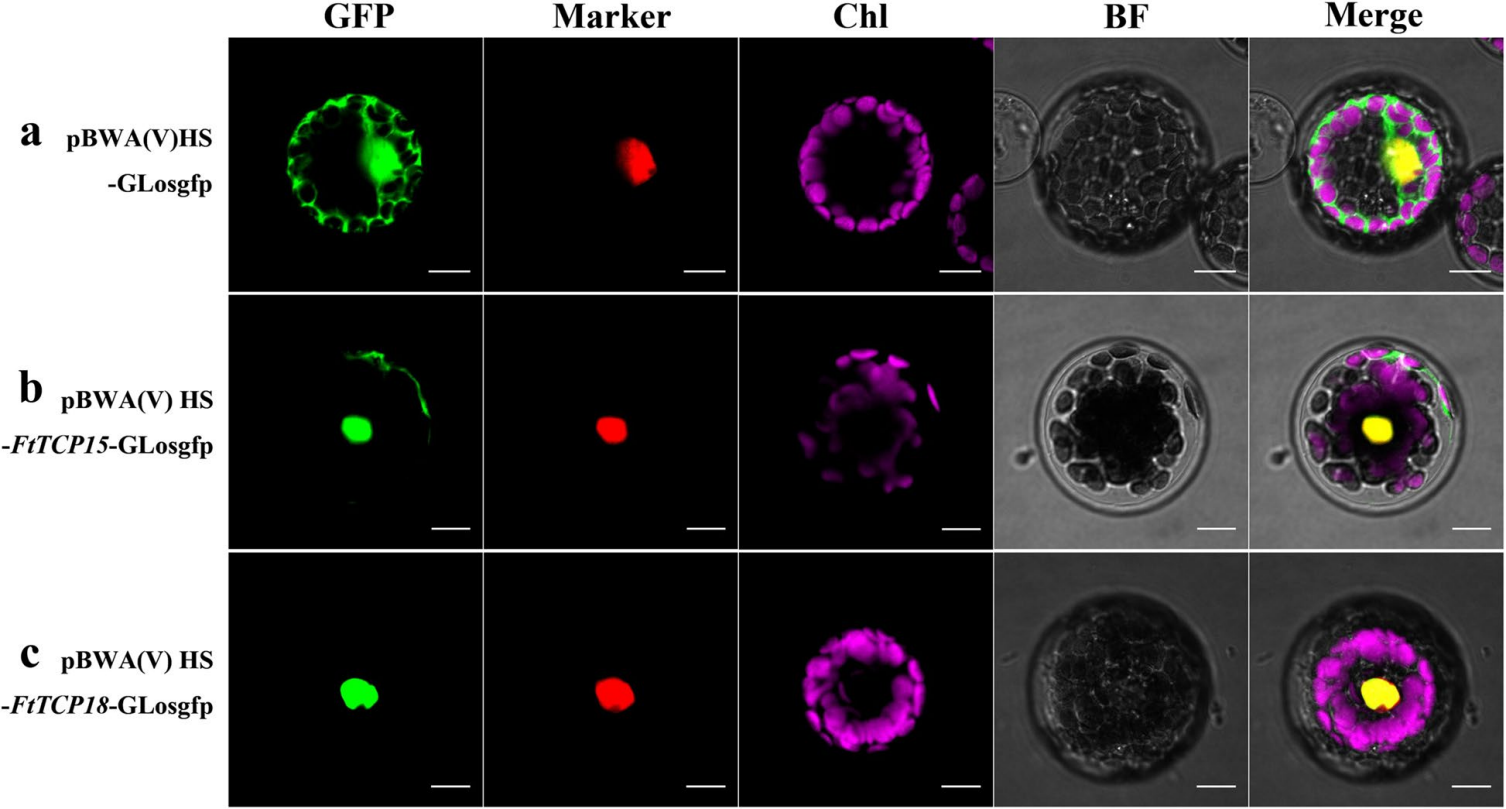
Subcellular localization analysis of *FtTCP15* and *FtTCP18*. **a** Localization results of pBWA(V)HS-GLosgfp empty plasmid as the control. **b** Colocalization of pBWA(V) HS-FtTCP15-GLosgfp with vacuolar membrane marker gene Attpk. **c** Colocalization of pBWA(V) HS-FtTCP18-GLosgfp with vacuolar membrane marker gene Attpk. GFP: Green fluorescent protein signal; Marker: Vacuolar membrane maker Attpk protein signal; Chl: Chlorophyll autofluorescence signal; BF: Bright field; Merge: Overlap of pictures. Bars = 10 μm

Transcription factors can regulate the transcription process of genes in the nucleus. To a certain extent, transcription factors can be determined by their subcellular localization. Figure 9 shows the subcellular localization analysis of the *FtTCP15* and *FtTCP18*. It is found that both *FtTCP15* and *FtTCP18* can be localized in the nucleus, which is consistent with the general understanding of the properties of transcription factors [40]. FtTCP15 also shows weak fluorescence signals in the cytoplasm, suggesting many exceptional cases for subcellular localization of transcription factors.

## Discussion

### *TCP* gene family in Tartary buckwheat is highly conserved

The phylogenetic analysis and sequence alignment of Tartary buckwheat, Arabidopsis, and rice indicate that all 26 *FtTCP*s can be classified into Class I (PCF) and Class II (CIN and CYC/TB1) (Fig. 2). Genes with close positions in the rootless phylogenetic tree have similar domains, which indicates that their functions are similar. For instance, the *FtTCP15* is closed to the *AtTCP10*, which is one of the members of miR319-regulated class II *TCP*s and regulates multiple physiological processes, including jasmonic acid (JA) biosynthesis [41]. The *OsTCP19* gene belonging to PCF (Class I) has been reported to play a role in modulating abscisic acid pathways to enhance the tolerance to drought and heat during the seedling and maturity stages [19]. The *FtTCP18* may have the same function.

Among the two subfamilies, the Class I subfamily has more genes without introns in structure. Moreover, the same group of *FtTCP* members displays relatively conserved motif compositions and a similar exon–intron organization (Fig. 3), indicating that the *FtTCP*s have close evolutionary relationships and similar functions to the *TCP* genes in plants. The similarity of gene structure provides further evidence for the credibility of subfamily classification. Introns can increase the length of genes and the frequency of gene recombination. In this study, 16 *FtTCP*s, including the *FtTCP15* and *FtTCP18,* have no introns, accounting for 62%. Genes with few introns tend to respond to stress more rapidly [42], and the introns-less genes of *FtTCP*s may facilitate the response to environmental changes. Additionally, specific conserved motifs are also one of the factors that contribute to the potential function. For example, Motif 5 appears twice in *FtTCP15* and may have a special significance for the function of *FtTCP15*.

Moreover, the expansion in the evolution of the *TCP* family in Tartary buckwheat is due to segmental duplications rather than tandem duplications (Fig. 4). This fact is consistent with previous research results in Arabidopsis, cotton [43], and grape [44]. The results show that segment duplication is the main driving force for the evolution and expansion of the *TCP* gene family, coinciding with the relatively high conservation of the *TCP* gene family. In addition, more orthologous gene pairs from the CIN group were identified among Tartary buckwheat, rice and Arabidopsis (Fig. 5), showing that *TCP* genes in these plant species originated from a common ancestor during evolution and the genes in the CIN group have the same function.

In conclusion, the lack of introns in gene structure and the conserved nature in evolution endow the functional similarity and stability of *TCP*s across species, which provides an important genetic basis for further studying the function of *TCP*s in the response of Tartary buckwheat to abiotic stresses.

### Cis-elements indicate the *FtTCP15* and *FtTCP18* play a unique role in the response of Tartary buckwheat to abiotic stresses

*TCP* transcription factors usually mediate the transmission of hormone signals, regulate the activity of phytohormone [45], and inhibit the promoting effect of gibberellin [46] and the biosynthesis of auxin [47] in the plant. Recent studies in Arabidopsis have suggested that members of the *TCP* family can form response modules with other genes to jointly regulate methyl jasmonate homeostasis [41], and thus may affect plant responses to abiotic stresses. Therefore, the *FtTCP*s can directly or indirectly affect cellular functions of plants, thereby affecting the plants’ growth, development, and response to abiotic stresses.

In this paper, the proportion of cis-elements responding to stress is 17% in Tartary buckwheat (Table S3, S4). The relatively high number of two cis-element promoters (anaerobic induction and anoxic specific inducibility) would result in the high expression of *TCP* family members in the anaerobic environment, such as WL. Typically, WL is probably caused by artificial irrigation, drainage, and excessive non-human rainfall. An oxygen-deficient state will occur when plants are in a flooded state for a long time [48]. These results provide insights into the molecular mechanism of the response of *FtTCP* genes, especially *FtTCP15* and *FtTCP18*, to WL (Fig. 7). Particularly, both *FtTCP15* and *FtTCP18* were significantly activated by WL and UV treatments (Fig. 2). Moreover, all light-responsive elements were found to be abundant in all *FtTCP*s. This result may be related to the biological clock regulation function of the *TCP* gene family previously reported [49]. In addition, we speculated that the light response elements are also related to the UV stress response. Ultraviolet radiation can affect plant growth and morphogenesis, especially in places with sufficient light, and high doses of UV-B, which can affect physiological and biochemical processes [31].

These results demonstrate that the *TCP* gene family may be involved in plant responses to abiotic stresses by regulating different biological processes such as direct response, hormonal regulation, and indirect response to abiotic stresses through morphogenesis. For example, the COM1 protein, which is homologous to the BDI1 *TCP* regulator in sorghum and Brachy podium, has been reported to adjust the shape of the spike inflorescence [50]*.*

### The *FtTCP*s act as important nodes in the interaction network in response to abiotic stresses

In recent years, several works have demonstrated that transcription factors could be promising targets for improving adaptability under environmental stress. In this work, 26 *FtTCP* genes were identified from the Tartary buckwheat genome. Under six abiotic stresses, the selected 12 genes in the root, stem, and leaf responded to different stress environments and had different expression patterns in different tissues and at different times. Among the 12 *FtTCP*s, five genes were activated by the specific stress, and the correlation coefficients of gene expression levels under abiotic stress were calculated (Fig. 7; Figure S1). The *TCP* family in soybean exhibited similar response trends under different abiotic stresses [51]. In this work, *FtTCP2* and *FtTCP8* showed the highest correlation of expression levels under abiotic stress and both of them were highly expressed in the stem (Fig. 8a). The *FtTCP18* participated in multiple stress responses. Additionally, *FtTCP15* was highly up-regulated under WL and UV environments, with the root, stem, and leaf expressed for 24 h.

Transcription factors, such as homo-dimers and hetero-dimers, often build combinations or interact with other proteins rather than acting alone to perform their complex biological functions [8]. Moreover, studies have shown that *TCP*s belonging to the CIN group can inhibit *MYB* gene-related transcripts and protein complexes at the transcription and protein levels [21], indicating that *TCP* members can form a regulatory network through interactions with other genes. The *TCP*s module, consisting of Class I and Class II TCP proteins, has been reported to manipulate important development processes, including hormone homeostasis in Arabidopsis [41]. In this study, *FtTCP*s have a high interaction frequency in the interaction network. For example, the nodes of *FtTCP15* and *FtTCP18* are 41 and 44, respectively. These results indicated that *FtTCP*s have an important regulatory function in the interaction network and play the regulatory role in different tissues of Tartary buckwheat.

### The regulation role of *FtTCP*s as transcription factors

Subcellular localization shows that *FtTCP18* is located in the nucleus (Fig. 9). Additionally, the determination of the subcellular localization of *FtTCP15* was in the nucleus and cytoplasm. Some studies also indicated that the subcellular localization of transcription factors is not in the nucleus or incompletely in the nucleus [52, 53]. There are many possible reasons for this phenomenon. Firstly, the results of subcellular localization analysis with different receptor materials are probably inconsistent. For example, *bHLH039* is mainly localized in the cytoplasm of tobacco leaf cells. In contrast, the nucleus and cytoplasm of Arabidopsis protoplasts have obvious signals [53]. Secondly, some treatments can change the location of transcription factors. As a transcription factor, *BZR1* is located in the cytoplasm without any treatment. After Brassinosteroids (BRs) treatment, *BZR1* is recruited into the nucleus because the original components in the cytoplasm have been transferred [52].

In conclusion, we proved that the *FtTCP* family plays a biological function as transcription factors in buckwheat by laboratory methods. For further study, various methods can be used to verify the transcriptional activity of *FtTCP*s. For example, a yeast experimental system can be used to analyze the transcriptional activation activity of NAC family members [54].

### Further studies on the function and molecular mechanism of *FtTCP15* and *FtTCP18* response to abiotic stresses in Tartary buckwheat are imperative

As an important pseudo-cereal crop, Tartary buckwheat will also face severe challenges to growth in the natural environment. As the global climate worsens, abiotic stress factors such as WL, UV, salt, dark, heat, cold and/or their combinations pose a serious threat to the growth, yield, and quality of crops. Additionally, *TCP* transcription factors can transform various endogenous and environmental signals and provide the most comfortable conditions for plant growth [55]. It is credible to hypothesize the manipulation of candidate *FtTCP* members in Tartary buckwheat with multiple stress adaptability.

In this study, *FtTCP15* and *FtTCP18* can be regarded as promising candidates for improving the adaptability of abiotic stress in Tartary buckwheat for their molecular characteristics, response to multiple abiotic stresses, and high correlation with other proteins. It is imperative to further study their function by molecular biology techniques. For example, the role of transcription factors in response to abiotic stresses can be determined by studying the binding of transcription factors and promoters using the dual-luciferase gene detection test and by studying the interaction of transcription factors with other proteins through subcellular colocalization tests. Conclusions about the analysis of the clear transcription network, identification of target genes, clarification of the connection with hormones and/or metabolic synthesis, and transcription modification [56] can elucidate the systemic functions of *FtTCP*s transcription factors in response to stress in Tartary buckwheat.

## Conclusions

Many studies have reported that the *TCP* gene family is vital to abiotic stress response in plants. In this work, 26 *FtTCP*s were identified in Tartary buckwheat and unevenly distributed on eight chromosomes. Further studies on evolutionary relationships, conservative motifs, gene structure, and cis-elements of the *FtTCP* gene family have been conducted. Under multiple abiotic stress treatments, the expression levels of the 12 selected *FtTCP*s were correlated, and the expression patterns of these genes tended to be spatio-temporal specific. The interaction network prediction of *FtTCP*s with many proteins indicated that *FtTCP*s are essential in the genetic manipulation response to abiotic stress. In addition, *FtTCP15* and *FtTCP18* are responsive to multiple abiotic stresses. The subcellular localization showed that both *FtTCP15* and *FtTCP18* localized in the nucleus, and the properties of *FtTCP*s as transcription factors are confirmed by laboratory methods. The results demonstrated the conserved nature in the evolution of the *TCP* gene family in Tartary buckwheat and suggested that the *TCP* gene family is functionally conserved in different species. The results also showed that the *TCP* gene family may be involved in plant responses to abiotic stresses by regulating different biological processes. Therefore, further studies on the functions of *FtTCP15* and *FtTCP18* by molecular biology techniques are feasible and imperative.

## Methods

### Bio-informatics data of *TCP* genes in Tartary buckwheat

In this study, two approaches were used to identify the *TCP* family in the Tartary buckwheat genome. The Hidden Markov Model (HMM) profile and the whole genome sequence information of Tartary Buckwheat were obtained from TBGP (Tartary Buckwheat Genome Project; http://www.mbkbase.org/Pinku1/). These sequences were then combined to compare the model with the TCP domain (PF03634) from the Pfam protein family database (http://pfam.xfam.org/) to ensure consistency. Two BLASTp methods were used to identify FtTCP members. First, all possible FtTCP proteins referring to TCP protein sequences were identified with BLASTp. Then, candidate FtTCP proteins containing the TCP domain were obtained by removing the redundant sequences using PFAM and SMART programs. Second, BLASTp in NCBI was used to verify whether all candidate FtTCPs were members of the TCP family. Finally, 26 *FtTCP*s were identified. As shown in Table S1, the coding sequence (CDS) length, isoelectric point (pI), and amino acid numbers were analyzed. Conserved domains for protein were analyzed using SMART (http://smart.embl-heidelberg.de/). The subcellular localization was predicted using the ExPasy website (http://web.expasy.org/protparam/).

### Genome-wide characteristic analysis of the *FtTCP* gene family

The distribution of *FtTCP*s on eight Tartary buckwheat chromosomes was obtained using CIRCOS software (Version 0.69).

Then, the TCP protein sequence was obtained from the UniProt database (https://www.uniprot.org). The full-length amino acid sequences and TCP domain sequences, 26 from Tartary buckwheat (*FtTCP*), 24 from Arabidopsis (*Arabidopsis thaliana* (L.) Heynh.) (*AtTCP*), and 22 from rice (*Oryza sativa* L.) (*OsTCP*), were used to generate an unrooted phylogenetic tree. The Arabidopsis TCP protein sequence was downloaded from The Arabidopsis Information Resources (TAIR) database (http://www.arabidopsis.org). The TCP protein sequences for multiple amino acid sequence alignments in three plants were analyzed, and the phylogenetic tree was built using the neighbor-joining (NJ) method of MEGA 7.0 Software, Geneious R11 with a BLOSUM62 matrix, and the Jukes Cantor substitution model. We set the global alignment with free end gaps and a bootstrap value of 1000.

The exon/intron structures of *FtTCP*s were generated with TBtools software [57]. The differences in the conserved protein motifs in Tartary buckwheat TCP proteins were compared using the protein conserved motif online search program MEME (http:/meme.nbcr.net/meme/intro.html). The motif breadth was set as 6 to 200 amino acids (aa). A total of 15 motifs (named Motif 1 to Motif 15) were selected for analysis (Table S2).

Multiple collinear scanning toolkits (MCScanX) with an E-value of 1e-10 were used to study the gene duplication events in *FtTCP* genes. In addition, the relationship of the *TCP* family between Tartary buckwheat and other plants, including Arabidopsis (*Arabidopsis thaliana* (L.) Heynh.), tomato (*Solanum lycopersicum* (L.) Mill.), soybean (*Glycine max* (L.) Merr.), apple (*Malus pumila* L.), rice (*Oryza sativa* L.) and maize (*Zea mays* (L.) Sp.), was analyzed using Dual Systeny Plotter software (Version 1.0 +) (https://github.com/CJ-Chen/TBtools).

The sequence regions 2000 bp upstream of *FtTCP* family members were extracted from the Tartary buckwheat genome as putative promoters. Then, the cis-acting elements on TBtools software (Version 1.0 +) [57] were predicted according to these putative promoters (Table S3; Table S4).

### Plant growth and treatments

The seeds of Tartary buckwheat were cultured in plastic pots in Guiyang, Guizhou Province, China. The cultivation environment was a 14/10 h photoperiod (daytime, 06:00–20:00) cycle with about 80% humidity at 22 °C. In order to analyze the expression profiles of *FtTCP* genes in roots, stems, and leaves in response to stress, 6-week-old seedlings were subjected to multiple abiotic stresses, including cold (low temperature, 4 °C), dark, heat (40 °C), salt (salinity, 200 mM NaCl) [34], UV radiation (70 µW/cm^2^, 220 V, 30 W), and WL (water inundating the surface of the earth with ddH_2_O). Under all treatments, samples were harvested with three biological replicates at 0, 2, and 24 h, then immediately frozen in liquid nitrogen, and stored at -80 °C for RNA extraction. Seedlings without stress treatment were the control. Moreover, seedlings grown in natural conditions were collected for tissue-specific analysis of genes with three biological replicates.

### RNA extraction, expression analysis by qRT-PCR, and interaction network prediction

In this work, to ensure that the response of genes contained in each subfamily to stress could be observed, 2 to 5 genes were randomly selected from each group (12 genes in total) for expression analysis. Total RNA was isolated from the sample using the RNAiso reagent Trizol (Invitrogen) according to the manufacturer's instructions. Then the potential genomic DNA contamination was removed from RNA using RNase-free DNase I (TaKaRa). The first-strand cDNA was synthesized using the Prime Script RT enzyme mix (Takara). Moreover, the QTower3G instrument (Analytik Jena AG) was used for the qPCR experiment, with each 10 µL amplification reaction containing 5 µL of 2 × PCR MIX (Roche), 0.1 µL of each primer (up 50 pM/µL, down 50 pM/µL), 3.8 µL of H_2_O, and 1 µL of cDNA template. The PCR cycle program included initial denaturation (95 °C/10 min) and 40 cycles of 94 °C for 10 s, 60 °C for 20 s, and 60 °C for 20 s. The gene-specific primers (Table S7) were designed based on the nucleotide sequence of the *TCP* gene in Tartary buckwheat using Primer 5.0 software (http://frodo.wi.mit.edu/). The Tartary buckwheat *FtH3* gene was employed as an internal reference sequence with the same program [58]. Three technical repeats were performed for each biological replicate, and the relative expression levels of each gene under abiotic stress were calculated by the 2^–∆∆Ct^ method [59] compared with the control gene. The relative expression levels for tissue-specific analysis of each gene were calculated by the 2^–∆Ct^ method. Finally, variance analysis and histogram were conducted on all data, and Duncan's multiple range test (*P* < 0.05; *n* = 3) was performed using SPSS (Version 25.0) and the Origin software (Origin Lab Corporation, Northampton, Massachusetts, USA) (Version 8.0) statistics program. Data correlation was analyzed using the RStudio (https://www.rstudio.com/) “corrplot” package. The analysis of heatmaps visualizing expression was performed using TBtools software (Version 1.0 +) [57].

The interaction network between the TCP family with known proteins and predicted proteins was predicted using the String (https://string-db.org/) for online protein-protein interaction prediction. The prediction included direct physical interactions between proteins and indirect protein functional relevance interactions. After downloading, relevant data of the prediction (Table S3; Table S4) were visualized using Cytoscape (Version 3.8.2) (https://cytoscape.org/download.html).

### Subcellular localization

According to the expression levels of genes under abiotic stress, *FtTCP15* and *FtTCP18* were found to be generally responsive to multiple abiotic stresses. Moreover, we are interested in further exploring the molecular mechanisms of *FtTCP15* and *FtTCP18.*

First, wild-type Colombian Arabidopsis seedlings were cultured in greenhouses at 25 °C for protoplast preparation. The first step: After 25 days of incubation (no bolting), some leaves were soaked in the enzymolysis solution (Cellulase R10, 1.5%; Macerozyme R10, 0.75%; Mannitol, 0.6 M; MES (pH 5.7), 10 mM; H_2_O, constant volume of 10 mL), and then left to stand at 24 °C for 4 h. The second step: The treated samples were filtered through a 40 μm strainer and centrifuged at 300 rpm for 3 min to remove the supernatant. Then samples were washed twice with 10 mL of pre-cooled W5 solution (NaCl (58.5), 154 mM; CaCl2 (111), 125 mM; KH_2_PO_4_ (136), 2 mM; MES, 2 mM; Glucose (180), 5 mM; H_2_O, constant volume to 100 mL) and centrifuged at 300 rpm at 4 °C for 3 min. The third step: 500 μL of MMG suspended solution (Mannitol, 0.4 M; MgCl_2_∙6H_2_O (203), 15 mM; MES, 4 mM; H_2_O, constant volume of 100 mL) was added to 100 μL of protoplasts and then observed under a 40 times microscope. The number of protoplasts per field should reach 20–40 to meet the requirements. The Cellulase R10, Macerozyme R10, Mannitol, MES (pH 5.7), and PEG4000 were provided by BIOSHARP (Labgic Co., Ltd, China); CaCl_2_, NaCl, KH_2_PO_4_, and MgCl_2_∙6H_2_O were purchased from Sinopharm Chemical Reagent Co., Ltd, China.

Second, the coding regions of *FtTCP15* and *FtTCP18* were amplified by PCR and fused to the pBWA(V)HS-ccdb-GLosgfp (Wuhan RiORUN Co., Ltd, China), which was modified by the pCAMBIA1302 expression vector. Using pBWA(V)HS-ccdb-GLosgfp as control, pBWA(V)HS-FtTCP15-GLosgfp and pBWA(V)HS-FtTCP18-GLosgfp were transferred into the Arabidopsis protoplasts prepared from leaves [60]*.* The 100 μL of protoplast suspension was mixed with a 20 μL plasmid of the target DNA plasmid. Simultaneously, PEG4000 solution (120 μL) equal to the total volume of DNA and protoplast was added, gently mixed, and left to stand at room temperature for 30 min. Finally, the reaction was terminated with 1 mL of W5 dilution. The reactants were centrifuged at 300 rpm for 3 min to collect protoplasts and remove the supernatant. After washing twice, 1 mL of W5 solution was added again and cultured at 28 °C for 24 h in the dark. After transformation, GFP fluorescence was observed under a laser scanning confocal microscope (Nikon C2-ER, Japan). All transient expression assays were repeated at least four times.

## Supplementary Information

**Additional file 1: Supplementaryfile 1: Table S1.** Detail informationdescription of the *TCP* gene family in Tartary buckwheat (*Fagopyrumtataricum *(L.) Gaertn.).

**Additional file 2: Supplementaryfile 2: Table S2.** The logo and name ofmotifs of *FtTCP*s in this study.

**Additional file 3: Supplementaryfile 3: Table S3.** The cis-elements of the *FtTCP*genome sequence (detail information).

**Additional file 4: Supplementaryfile 4: Table S4. **The cis-elements of the *FtTCP*genome sequence (data analysis).

**Additional file 5: Supplementaryfile 5: Figure S1.** The correlation analysisof the gene expression of 12 selected *FtTCP*s under different abioticstresses in this study.

**Additional file 6: Supplementaryfile 6: Table S5.** The interaction networkdata of *FtTCP*s from STRING in this study.

**Additional file 7: Supplementaryfile 7: Table S6.** The interaction network of*FtTCP*s in this study.

**Additional file 8: Supplementaryfile 8: Table S7.** The primers used forqRT-PCR of *FtTCP*s in this study.

## Data Availability

The whole *Fagopyrum tataricum* genome sequence information was obtained from the website (http://buckwheat.kazusa.or.jp/index.html), which is open to all researchers. The datasets supporting the conclusions of this article are included in the article and additional files. We have not used KEGG Pathway Database.

## Abbreviations

ABA: Abscisic Acid
ABRE: Abscisic Acid Response Element
ARE: Anaerobic
CDS: Coding Sequence
CIN: Cincinnata
CYC: Cycloidea
GFP: Green Fluorescent Protein
ID: Identity
JA: Jasmonic Acid
LTR: Low-Temperature Responsiveness
MBS: Myeloblastosis Binding Site
MCScanX: Multiple Collinear Scanning Toolkits
MEGA: Molecular Evolutionary Genetics Analysis
Me-JA: Methyl- Jasmonate
MEME: Multiple Expectation Maximizations for Motif Elicitation
MES: MES hydrate (C6H13NO4S)
MMG: Monomycolylglycerol solution
Mw: Molecular weight
NAC: No apical meristem & Ataf1/2 & cup-shaped cotyledon
PCF: Proliferating Cell Factors
PEG: Polyethylene Glycol
pI: Isoelectric point
PL: Length of protein
qRT-PCR: Quantificational Real-Time Polymerase Chain Reaction
SMART: Simple Modular Architecture Research Tool
TB1: Teosinte Branched 1
TBGP: Tartary Buckwheat Genome Project
TCP: Teosinte branched 1 & Cycloidea & Proliferating Cell Factors
TF: Transcription Factor
UV: Ultraviolet
UV-B: Ultraviolet-Biological
W: Predicted subcellular location
WUN: Wound-Responsive
WL: Water Logging
