# Supplementary material for: Systematic analysis and expression profiles of TCP gene family in Tartary buckwheat (Fagopyrum tataricum (L.) Gaertn.) revealed the potential function of FtTCP15 and FtTCP18 in response to abiotic stress
Source: BMC Genomics. 2022 Jun 2;23:415. doi: 10.1186/s12864-022-08618-1 (PMC9164426; doi:10.1186/s12864-022-08618-1)

Figure legend

Figure S1. The correlation analysis of the gene expression of 12 selected FtTCPs under different abiotic stresses in this study. Data correlation was analyzed using the RStudio "corrplot" package.

Figure S1.

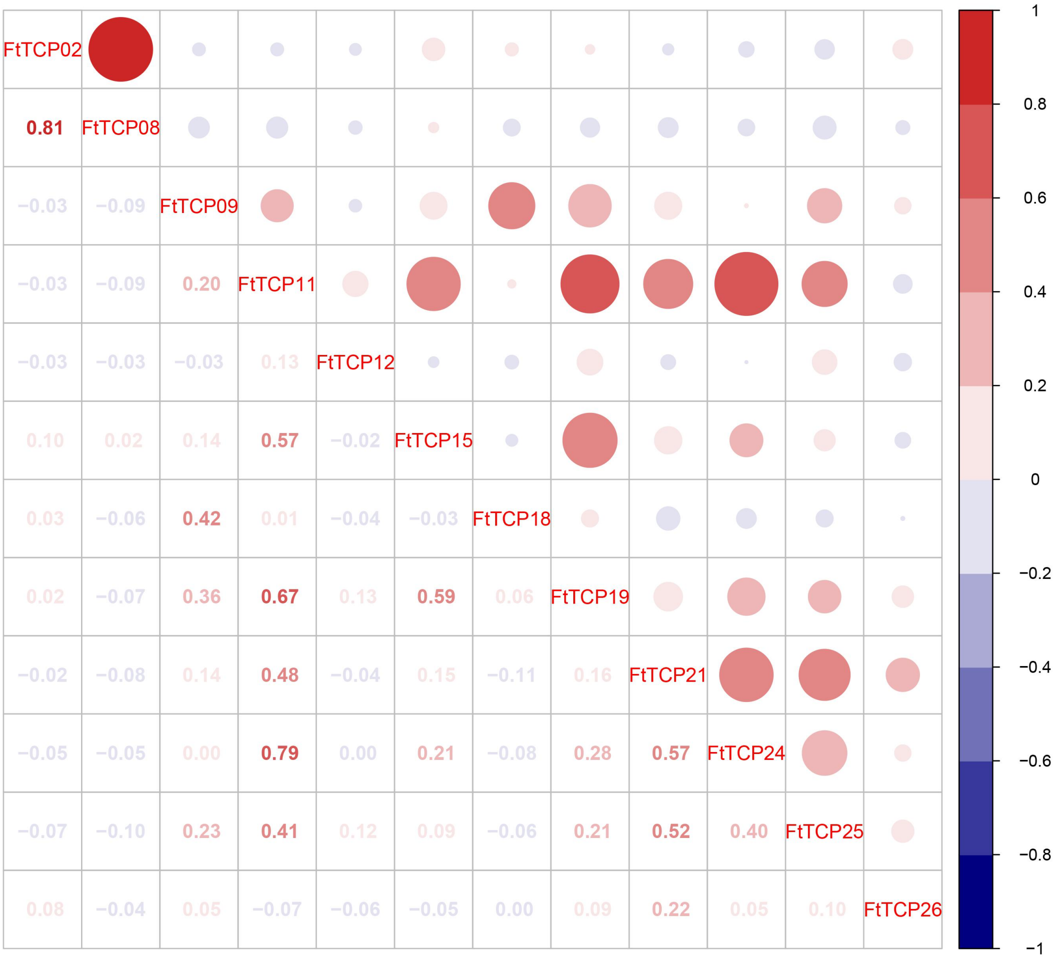

Supplement: Supplementary file 5 — Additional file 5: Supplementaryfile 5: Figure S1. The correlation analysisof the gene expression of 12 selected FtTCPs under different abioticstresses in this study. [file 12864_2022_8618_MOESM5_ESM.pdf]
